# Vitamin D_3_ eradicates *Helicobacter pylori* by inducing VDR-CAMP signaling

**DOI:** 10.3389/fmicb.2022.1033201

**Published:** 2022-12-08

**Authors:** Ye Zhang, Chunya Wang, Li Zhang, Jie Yu, Wenjie Yuan, Lei Li

**Affiliations:** ^1^Department of Gastroenterology, Affiliated Hospital of Weifang Medical University, Weifang, China; ^2^Department of Hepatobiliary Surgery, The First Affiliated Hospital, Kunming Medical University, Kunming, China

**Keywords:** 1α, 25-dihydroxyvitamin D_3_, *H. pylori*, vitamin D receptor, cathelicidin antimicrobial peptide, inflammation

## Abstract

**Background:**

Vitamin D_3_ [VitD_3_, 1,25 (OH)_2_D_3_] is known to have immunomodulatory and anti-microbial properties; however, its activity against *Helicobacter pylori* is unclear. In this study, we established *H. pylori* infection models in wild-type and VitD_3_ receptor (VDR) knockdown mice and analyzed the effects of VitD_3_ and their underlying mechanisms.

**Methods:**

VDR^+/+^ and VDR^+/–^ mice were intragastrically infected with the *H. pylori* SS1 strain. After confirmation of *H. pylori* infection, mice were treated with different doses of VitD_3_. The infection levels in stomach tissues were quantified using the colony-forming assay, and the expression levels of the VDR and cathelicidin antimicrobial peptide (CAMP) in the gastric mucosa were analyzed by immunohistochemistry and western blotting.

**Results:**

The gastric mucosa of VDR^+/–^ mice was more susceptible to *H. pylori* colonization and had lower levels of VDR and CAMP expression than that of VDR^+/+^ mice. *H. pylori* infection upregulated VDR and CAMP expression in the stomach of both wild-type and mutant mice, and VitD_3_ treatment resulted in further increase of VDR and CAMP levels, while significantly and dose-dependently decreasing the *H. pylori* colonization rate in both mouse groups, without affecting blood calcium or phosphorus levels.

**Conclusion:**

Our data indicate that oral administration of VitD_3_ reduces the *H. pylori* colonization rate and upregulates VDR and CAMP expression in the gastric mucosa, suggesting a role for VitD_3_/VDR/CAMP signaling in the eradication of *H. pylori* in the stomach. These findings provide important insights into the mechanism underlying the anti-*H. pylori* activity of VitD_3_ and should be useful in the development of measures to eradicate *H. pylori*.

## Introduction

*Helicobacter pylori* colonizes the gastric epithelium of approximately half of the world’s population and is classified as a class I carcinogen by the World Health Organization (Malfertheiner et al., 2017). Both sanitary conditions and socio-economic status are important factors in the prevalence of *H. pylori* infection, which is higher in developing than in developed countries. The pathogenic activity of *H. pylori* can result in such diseases as chronic gastritis and peptic ulcer, and individuals carrying the bacteria for many years have an increased risk of gastric cancer and gastric mucosa-associated lymphoid tissue lymphoma. Furthermore, in users of non-steroidal anti-inflammatory drugs, *H. pylori* infection may increase the risk of gastric bleeding (Mitchell and Katelaris, 2016). *H. pylori* may also play a role in many extragastric diseases, including idiopathic thrombocytopenic purpura, unexplained iron deficiency anemia, and vitamin B12 deficiency (Huang et al., 2010). The eradication of *H. pylori* can effectively prevent the occurrence of these pathological conditions; however, it is difficult for the host to clear the infection through the innate immune system. Proton pump inhibitor (PPI)-based triple therapy was once the first-line approach to *H. pylori* eradication (Malfertheiner et al., 2007), but the widespread use of antibiotics has led to the emergence of single- and multiple-drug-resistant *H. pylori* strains, making its eradication more difficult (Malfertheiner et al., 2012; Savoldi et al., 2018). At present, quadruple therapy is considered to be an effective alternative regimen, especially in developing countries where the population has high resistance to clarithromycin or metronidazole (Kim et al., 2014). Our previous research indicates that bismuth in a compound preparation, Wei Bi Mei, has higher efficacy and safety in eradicating *H. pylori* compared to commonly used bismuth medicines: it can significantly reduce *H. pylori* colonization, while showing the fastest clearance and the lowest accumulation rates in organs (Li et al., 2018). However, new drug-resistant strains continue to emerge, while safe and effective vaccines are still under development (Stubljar et al., 2018; Walduck and Raghavan, 2019). Therefore, there is an urgent need for new antibacterial agents with fewer adverse effects to improve on the current status of *H. pylori* eradication.

Vitamin D (VitD) is a steroid hormone necessary for bone mineralization. Obtained from food or through solar exposure, VitD is inactive and is transported to the liver, where 25-hydroxyvitamin D (calcidiol) is produced through the activity of microsomal VitD-25-hydroxylase and is then either stored in the liver or released into the bloodstream. In the kidney, calcidiol is catalyzed by mitochondrial 1a-hydroxylase (CYP27B1), produced by the renal proximal tubule epithelial cells, into 1α,25-dihydroxyvitamin D_3_ (VitD_3_), the activated hormonal form of VitD (Reeve et al., 1983) which regulates calcium and phosphorus absorption in the intestine, mobilizes bone calcium, and maintains the balance of calcium and phosphorus in serum (Christakos et al., 2012).

Now, it is increasingly recognized that VitD_3_ is not only related to the diseases of the skeletal system but is also associated with many other physiological processes in the human body. VitD_3_ exerts its functional effects through binding to the VitD receptor (VDR), a transcription factor that belongs to the nuclear receptor superfamily and is found in almost all cell types of the human organism (Carlberg, 2014). The VDR not only controls the expression of genes related to mineral metabolism but also interacts with other intracellular signaling pathways such as those regulating immune reactions, cell cycle progression, and apoptosis. The effects of VitD_3_ on immune responses to bacterial infections, especially to *Mycobacterium tuberculosis*, have been documented in many studies. VitD3 has been shown to promote autophagy in *M. tuberculosis*-infected macrophages and induce the activation of Toll-like receptors (TLRs), thus inducing VDR, CYP27B1, and CYP27B1 expression and the synthesis of biologically active VitD_3_; the latter in turn binds to the VDR and upregulates the expression of cathelicidin antimicrobial peptide (CAMP), ultimately enhancing immune response and promoting the eradication of intracellular *M. tuberculosis* (Hmama et al., 2004; Schauber et al., 2007; Hewison, 2011). VitD can also reduce the incidence of respiratory tract infections. A previous study found that serum VitD levels were negatively correlated with the rate of recent upper respiratory infections among 19,000 participants, who had been followed for an average of more than 12 years (Ginde et al., 2009). In a cohort study including 800 participants, it was found that the number of days of absence from duty due to respiratory infection was significantly higher for the participants with serum VitD levels <40 nmol/L than for the control group (Laaksi et al., 2007). As VitD_3_ is a direct inducer of CAMP, which is known to mount immune response against a variety of pathogenic microorganisms, including gram-positive and gram-negative bacteria, viruses, and fungi (Liu et al., 2006; Wang et al., 2019), we hypothesize that the VitD_3_–CAMP axis may be involved in the immune defense against *H. pylori*.

Accumulating evidence indicates that VitD is associated with the risk of *H. pylori* infection and failure of its eradication. Specifically, serum VitD levels are higher in patients successfully treated for *H. pylori* infection than in those with treatment failure (Yildirim et al., 2017). Serum VitD levels also have a significant positive correlation with *H. pylori* infection in uremic patients (Nasri and Baradaran, 2007). VitD deficiency can promote the development of *H. pylori*-related chronic gastritis and increase the severity of gastric mucosal damage, whereas VitD supplementation can improve disease status (Zhang et al., 2016). It has been documented that *H. pylori* infection in children is significantly associated with VitD deficiency (Gao et al., 2020) and that its prevalence in elderly patients is decreased by VitD supplementation. Cumulatively, these findings indicate that VitD administration may improve the efficiency of *H. pylori* eradication and reduce drug-related adverse effects.

It has been shown that in human GES-1 cells, the expression of the VDR and CAMP is increased after *H. pylori* infection, whereas in mice, the inhibition of VDR expression leads to significant downregulation of CAMP mRNA and protein expression but upregulation of inflammatory factors (Guo et al., 2014). A previous study indicates that compared with wild-type mice, CAMP knockout mice have increased susceptibility to *H. pylori* colonization of the gastric mucosa and aggravated mucosal inflammation, whereas supplementation with exogenous CAMP can reduce *H. pylori* colonization and inflammation in the mucosa and decrease the production of inflammatory cytokines (Zhang et al., 2013). These results suggest that CAMP plays an important role in the prevention of gastric mucosa colonization by *H. pylori*.

In this study, we tested a hypothesis that the VDR/CAMP pathway may be involved in the inhibitory effect of VitD_3_ on *H. pylori* infection. For this test, we established *H. pylori* infection models in C57BL/6J wild-type and VDR knockdown mice and investigated the effect of VitD_3_ gavage. Our findings indicate that VitD_3_ can, in a concentration-dependent manner, eradicate *H. pylori* and induce the expression of VDR and CAMP *in vivo*, suggesting the mechanism underlying the anti-*H. pylori* activity of VitD_3_.

## Materials and methods

### Bacterial culture and strain adaptation

The *H. pylori* Sydney strain 1 (SS1) (kindly provided by Professor Chun-Jie Liu, Academy of Military Medical Sciences of the Chinese People’s Liberation Army (PLA), Beijing, China) was stored at −80°C. After thawing at room temperature, the bacterial suspension was dropped onto Campylobacter Base Agar plates containing three antibiotics (0.38 mg/L polymyxin B, 10 mg/L vancomycin, and 2 mg/L amphotericin B) and cultured for 36–72 h at 37°C under microaerobic conditions (5% O_2_, 10% CO_2_, and 85% N_2_). A small amount of cultured *H. pylori* was picked, evenly spread on slides dripped with Double Distilled Water, dried, and stained with flagellar staining solution (alkalescent carbolfuchsin; DM0031, Beijing Leagene Biotechnology Co., Ltd., Beijing, China) to assess bacterial growth status. Bacterial suspensions were prepared by adding sterile saline to *H. pylori*-containing plates and collecting for subsequent use.

Specific-pathogen-free (SPF) C57BL/6J mice (10-week-old males weighting 19–22 g) were purchased from Beijing Vital River Laboratory (Beijing Vital River Laboratory Animal Technology Co., Ltd., Beijing, China) and housed in the Animal Experiment Center of the Chinese Center for Disease Control and Prevention under a 12:12 h light-dark cycle in a standard environment of 23 ± 2°C and 50–60% relative humidity; distilled water and standard sterile mouse feed (Beijing Keao Xieli Feed Co., Ltd., Beijing, China) were provided *ad libitum*. The adapted *H. pylori* strains capable of mouse colonization were obtained by serial passage *in vivo* and used to establish infection models. Mice were administered bacterial suspension [10^8^ colony forming units (CFU)/L] orally and euthanized 4 weeks later. Stomachs were removed, washed, and cut longitudinally into halves along the greater curvature; one half of the gastric mucosa was suspended, transferred to plates, and cultured for 36–72 h to reveal the presence or absence of *H. pylori*, whereas the other half was used for direct smear microscopy. After confirmation of successful infection, the *H. pylori* isolate with the highest number of colonies was selected as the first-generation adapted strain. The next-generation strain was obtained after infecting new mice with the first-generation strain as described above. The procedure was repeated until stable infection in mice was achieved. All animal experiments were approved by the Laboratory Animal Ethics Committee of Beijing Friendship Hospital, Affiliated to Capital Medical University, and were performed in accordance with institutional guidelines.

### Mouse models of *Helicobacter pylori* infection

Before infection, mice were fasted for 12 h and deprived of water for 4 h; then, they received 300 μL of 3% NaHCO_3_ by gavage to increase the pH in the stomach. Mice were then divided into control and *H. pylori* infection groups and intragastrically inoculated with 300 μL of sterile saline or the suspension of the adapted *H. pylori* strain (10^8^ CFU/L), respectively. Inoculation was performed twice in each subject, once 30 min after NaHCO_3_ treatment and once after 4 h. Mice were allowed free access to food and water 2 h after the last dose and were fed normally until the end of the experiment. Three months later, fecal genetic testing was performed to confirm *H. pylori* infection in the gastrointestinal tract. Mice were euthanized by spinal cord dislocation, and their gastric tissues were removed and stained with hematoxylin–eosin to analyze the inflammatory response. Warthin-Starry staining (SBJ-0548, Nanjing SenBeiJia Co., Ltd., Nanjing, China) and a *H. pylori* rapid detection kit (HPUT-H104, Fujian Sanqiang Biochemical Co., Ltd., Fujian, China) were used to observe *H. pylori* colonization.

### VitD_3_ intervention

Male 3–12 week-old C57BL/6J mice of the wild type (VDR^+/+^) and VDR knockout homozygous type (VDR^–/–^; B6.129S4-*Vdr^TM^*^1*Mbd*^/J, SPF) were purchased from Jackson Laboratory (Bar Harbor, ME, USA). VDR knockdown (VDR^+/–^) mice were obtained by *in vitro* fertilization in Beijing Biocytogen Co., Ltd.

VDR^+/+^ and VDR^+/–^ mice were randomly divided into five groups (*n* = 6 mice per group): control and *H. pylori* infection (HP) groups, and three HP + VitD_3_ groups. Mice in the control group were intragastrically inoculated with sterile saline and those in HP/HP + VitD_3_ groups with *H. pylori* suspension as described above. After 3 months, the control and HP groups were orally administered equal volumes (300 μL) of corn oil, whereas the three HP + VitD_3_ groups were orally administered 0.1, 0.4, and 1.6 μg/kg of 1α,25-Dihydroxyvitamin D_3_ (D1530, Sigma Aldrich, St Louis, MO, USA) and designated as HP+VitD_3_*1, HP+VitD_3_*4, and HP+VitD_3_*16, respectively. All treatments were performed once a day for 14 consecutive days. Then, blood was collected from the eye, and mice were euthanized by spinal cord dislocation. Gastric tissue was removed under sterile conditions, washed, and cut longitudinally into halves along the greater curvature. One half was fixed in 10% formalin, embedded in paraffin, cut into 4 μm-thick sections, baked for 1 h at 60°C, deparaffinized in xylene, and rehydrated in graded ethanol for subsequent staining. The other half was used for the colony-forming assay and other tests.

### Warthin-starry staining

Deparaffinized tissue sections were washed three times for 1 min with distilled water, incubated in acidic silver nitrate solution for 1 h in the dark at 56°C, and immersed in Warthin-Starry solution for 3–8 min. After soaking in distilled water at 56°C and rinsing once with distilled water, sections were dehydrated in 100% ethanol, cleared with xylene for transparency, sealed with neutral gel, and analyzed by light microscopy. *H. pylori* appeared tan or black on a light yellow background.

### Rapid urease reaction

Fresh gastric mucosal tissue was placed into the liquid medium of the enzyme-labeled strip (enzymatic reaction solution) from the *H. pylori* detection kit, according to the manufacturer’s instructions, and incubated at 10–30°C for 5 min. The results were visually observed and interpreted according to color change of the liquid at the edge of gastric mucosal tissue: yellow (no chromogenic reaction) was treated as negative and light or rose red as positive for *H. pylori*.

### Determination of serum calcium and phosphorus levels

Blood samples were centrifuged at 3,000 rpm for 10 min, and about 100–300 μL of supernatant was collected to measure serum calcium and phosphorus contents using an automatic biochemical analyzer.

### Colony-forming assay

The level of *H. pylori* infection in mouse gastric tissue was quantified by the colony-forming assay. Approximately half of the stomach tissue was weighed, homogenized in 1 mL Brucella broth, serially 10-fold diluted, and spread on agar plates containing Campylobacter Base Agar, 10% fetal calf serum, 0.38 mg/L polymyxin B, 10 mg/L vancomycin, 2 mg/L amphotericin B, 5 mg/L trimethoprim, and 50 mg/L bacitracin (Merck KGaA, Darmstadt, Germany). After 72 h of incubation, the number of colonies per plate and CFUs per gram of stomach tissue were calculated.

### Immunohistochemistry

Paraffin sections were rehydrated through graded alcohol solutions and washed with distilled water. Antigen retrieval was performed by heating sections in 0.01 M sodium citrate buffer (pH 6.0) in a pressure cooker at 130°C for 3 min, and endogenous peroxidase activity was quenched by incubation in 3% hydrogen peroxide for 15 min. Then, tissue sections were incubated with anti-VDR C-20 or anti-CAMP antibodies (Santa Cruz Biotechnology, Santa Cruz, CA, USA) at 4°C overnight. After washing in PBS three times, sections were incubated with horseradish peroxidase-conjugated secondary anti-rabbit Ig (ZSGB-BIO, Beijing, China) for 1 h at room temperature, washed, and immersed in diaminobenzidine (ZSGB-BIO) for 1–2 min to develop color reaction. Finally, sections were counterstained with hematoxylin, dehydrated, and mounted in resin mounting medium.

### Western blotting

Total protein was extracted from gastric mucosal tissue using RIPA lysis buffer, and protein concentration was quantified using the Pierce BCA Protein Quantification kit (Thermo Fisher Scientific, Waltham, MA, USA). Proteins were separated by SDS/PAGE in 10% gels and transferred to polyvinylidene fluoride (PVDF) membranes, which were then blocked with 5% non-fat milk for 3 h at room temperature and incubated with antibodies against VDR (Santa Cruz Biotechnology, Santa Cruz, CA, USA), CAMP (Santa Cruz Biotechnology, Santa Cruz, CA, USA), or β-actin (Sigma, USA) at 4°C overnight. After three 10 min washes with Tris-buffered saline containing 0.1% Tween 20, the membranes were incubated with horseradish peroxidase-conjugated secondary antibodies at room temperature for 1 h, and signals were developed using the enhanced chemiluminescence kit (Bio-Rad, California, USA).

### Statistical analysis

The data are presented as the mean ± standard deviation (SD). Differences between groups were assessed by analysis of variance and standalone *t*-test. *P* < 0.05 was considered to indicate statistical significance.

## Results

**FIGURE 1:**
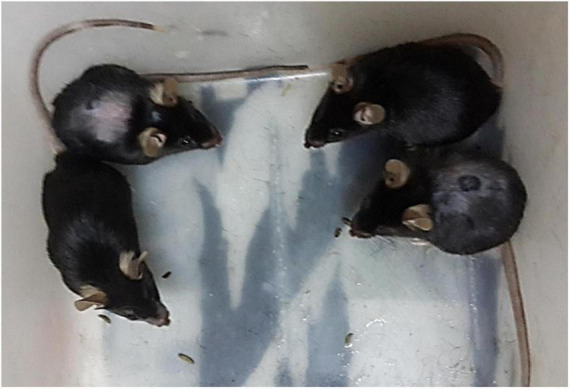
Mouse strains. VDR^+/–^ C57BL/6J mice (upper left and lower right) and VDR^+/+^ C57BL/6J mice (lower left and upper right).

The mouse tail genotype test showed that VDR^+/–^ C57BL/6J mice were successfully created and met the requirements of the experiment. Compared with wild-type VDR^+/+^ C57BL/6J mice, VDR^+/–^ C57BL/6J mice had less hair in the back, clearly showing the skin (Figure 1).

### Characteristics of the *Helicobacter pylori* Sydney strain 1 strain cultured *in vitro*

**FIGURE 2:**
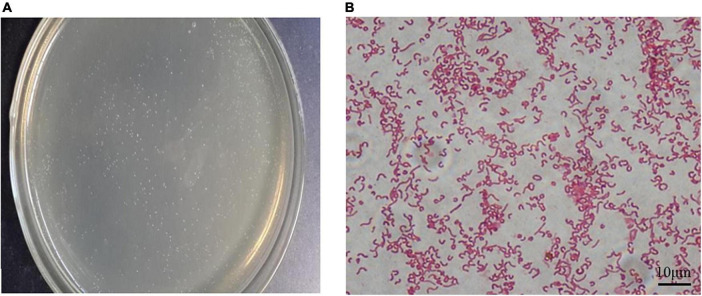
Cultivation of the *Helicobacter pylori* Sydney strain 1 (SS1) strain *in vitro*. **(A)** Typical white needle tip colonies could be seen on the plates after 72 h of incubation. **(B)** Bacterial flagella were observed after alkalescent carbolfuchsin staining. Scale bar = 10 μm.

After 72 h of culture, the resuscitated *H. pylori* SS1 strain formed typical spiculate translucent colonies (Figure 2A). In most smears, morphologically intact *H. pylori*, typically with flagella, were revealed after carbolfuchsin staining (Figure 2B), indicating good activity, and strong colonization ability of the adapted strain.

### *Helicobacter pylori* successfully colonized mouse stomachs

**FIGURE 3:**
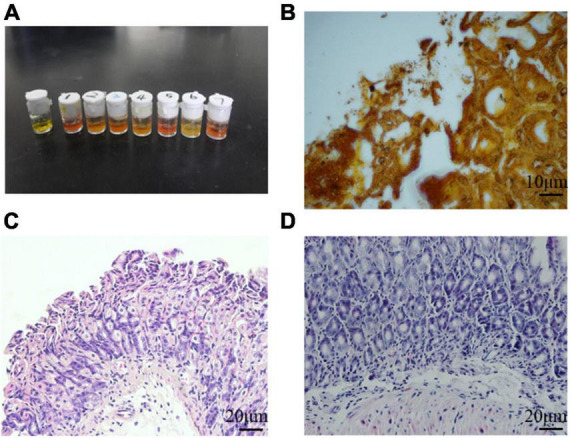
*Helicobacter pylori* successfully colonized mouse stomach tissue. **(A)** Rapid urease reaction showed positive results in infected mice and negative results in control mice. **(B)** Several black rod-shaped structures were observed after Warthin-Starry silver staining. Scale bar = 10 μm. **(C,D)** Hematoxylin-eosin staining of the gastric mucosa of uninfected **(C)** and infected **(D)** mice. Chronic gastric mucosal inflammation could be observed in the infected group. Scale bar = 20 μm.

It is known that *H. pylori* has urease genes and can produce large amounts of urease, which is necessary for its colonization of the human gastric mucosa and is used for routine diagnosis of *H. pylori* infection. Three months after *H. pylori* intragastric administration, the urease reaction test was positive in infected mice and negative in control mice (Figure 3A), indicating *H. pylori* colonization of gastric tissues. Several black rod-shaped structures were observed after Warthin-Starry silver staining (Figure 3B), confirming that *H. pylori* was present in the gastric mucosa. Compared with the gastric tissue of uninfected mice (Figure 3C), that of infected mice showed massive lymphocyte infiltration and increased presence of inflammatory cells (Figure 3D). These results indicated that the one-time oral gavage of the *H. pylori* SS1 strain resulted in the successful establishment of a mouse model of *H. pylori* infection, which produced chronic gastric inflammation after 3 months.

### *Helicobacter pylori* eradication efficacy of VitD_3_ in infected mice

**FIGURE 4:**
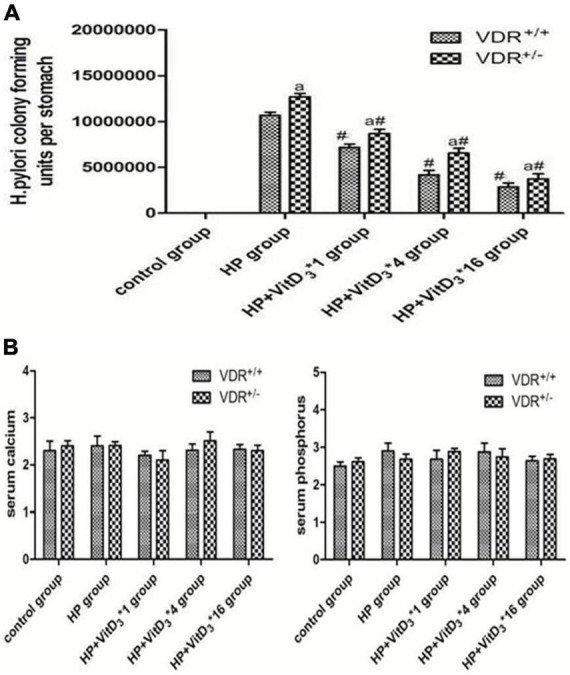
*Helicobacter pylori* eradication efficacy of VitD_3_ at different doses in infected mice. **(A)** Colony-forming assay results showing increased susceptibility of VDR^+/–^ mice to *H. pylori* colonization compared to VDR^+/+^ mice (*P* < 0.05). In both mouse strains, *H. pylori* colonization was significantly reduced after VitD_3_ administration and *H. pylori* numbers decreased in a dose-dependent manner (*n* = 6; ^#^*P* < 0.05 vs. HP group and *^a^P* < 0.05 vs. VDR^+/+^ group). **(B)** Serum calcium and phosphorus levels were comparable in VDR^+/+^ and VDR^+/–^ mice and remained in the normal range after VitD_3_ administration (*n* = 6; *P* > 0.05). HP, *H. pylori* infection.

The results of the colony-forming assay performed 3 months after intragastric administration of *H. pylori* indicated that VDR^+/–^ mice were more susceptible to *H. pylori* colonization than VDR^+/+^ mice (*P* < 0.05, Figure 4). In both wild-type and mutant mice, VitD_3_ administration significantly reduced *H. pylori* colonization compared with corn oil-treated control groups, and the number of *H. pylori* showed a gradual dose-dependent decrease (Figure 4A). There were no significant changes in food intake or body weight throughout the experiment, and all mice showed no abnormalities. After VitD_3_ intervention, serum calcium and phosphorus concentrations were still in the normal range in all mice (Figure 4B, *P* > 0.05).

### VitD_3_ upregulated VitD3 receptor expression in the mouse gastric mucosa

**FIGURE 5:**
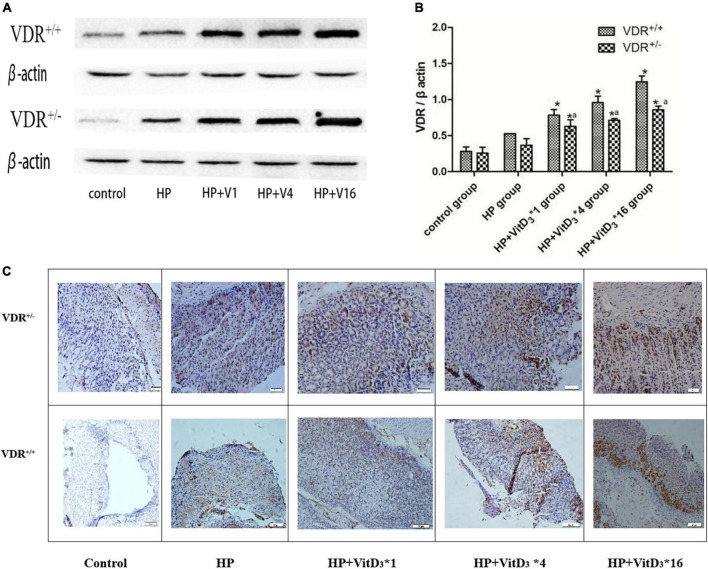
VitD_3_ increased VitD_3_ receptor (VDR) protein expression in mouse gastric tissues. **(A)** Western blotting analysis revealed a significant, dose-dependent increase of VDR expression by VitD_3_ in mouse gastric tissues. VDR protein expression was consistently lower in VDR^+/–^ mice than in VDR^+/+^ mice after VitD_3_ treatment (*n* = 6; *P* < 0.05). **(B)** Histogram showing quantitative analysis of the results presented in panel **(A)**. **(C)** Immunohistochemistry analysis of VDR expression in gastric tissues of infected mice treated or not with different doses of VitD_3_. *P < 0.05 vs. HP group and *^a^P* < 0.05 vs. VDR^+/+^ group. HP, *Helicobacter pylori* infection. Upper row, scale bar = 20 μm. Bottom row, scale bar = 50 μm.

To further investigate the mechanism of *H. pylori* clearance by VitD_3_, we analyzed VDR expression in control and infected mice, treated or not with VitD_3_. VDR protein expression was significantly upregulated in the gastric mucosa of VitD_3_-treated mice compared with that of corn oil-treated mice, and the effect was dose-dependent (*P* < 0.05; Figures 5A,B). Furthermore, after VitD_3_ treatment VDR protein expression in VDR^+/–^ mice was consistently weaker than that in VDR^+/+^ mice (*P* < 0.05). Immunohistochemistry analysis showed that the intensity of VDR staining increased with the VitD_3_ dose (Figure 5C), confirming the upregulation of VDR expression by VitD_3_. Collectively, these results indicate that VitD_3_ can induce VDR expression *in vivo*.

### VitD_3_ stimulated cathelicidin antimicrobial peptide expression in the mouse gastric mucosa

**FIGURE 6:**
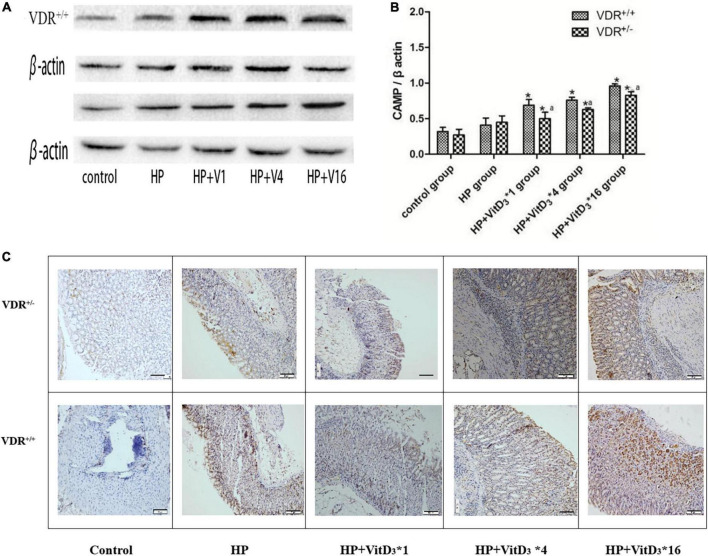
VitD_3_ upregulated cathelicidin antimicrobial peptide (CAMP) expression in the mouse gastric mucosa. **(A)** CAMP expression in the gastric mucosa was analyzed by western blotting. VitD_3_ gavage increased CAMP protein expression in a dose-dependent manner. The CAMP expression level was consistently lower in VDR^+/–^ than in VDR^+/+^ mice (*n* = 6; *P* < 0.05). **(B)** Histogram showing quantitative analysis of the results presented in panel **(A)**. **(C)** Immunohistochemistry analysis showing VitD_3_ dose-dependent increase of CAMP staining intensity in the mouse mucosa. *P < 0.05 vs. HP group and *^a^P* < 0.05 vs. VDR^+/+^ group. HP, *Helicobacter pylori* infection. Scale bar = 50 μm.

Cathelicidin antimicrobial peptide has a broad spectrum of antibacterial activities and can induce immune responses to a variety of pathogenic microorganisms. As VitD_3_ has been shown to upregulate CAMP expression, we hypothesized that CAMP could be involved in the clearance of *H. pylori* through the VitD_3_–VDR interaction. In agreement with the VDR expression results described above, the intragastric administration of VitD_3_ resulted in significant dose-dependent upregulation of CAMP expression in the gastric mucosa of *H. pylori*-infected mice, compared with that in the control HP group (*P* < 0.05; Figures 6A,B). CAMP expression was consistently lower in VDR^+/–^ mice than in VDR^+/+^ mice (*P* < 0.05). These results were consistent with the immunohistochemistry analysis (Figure 6C). Thus, CAMP may be involved in the anti-*H. pylori* activity of VitD_3_.

## Discussion

In this study, we bred VDR knockdown (VDR^+/–^) mice and compared their reaction to *H. pylori* infection and VitD_3_ administration with that of wild-type (VDR^+/+^) mice. Treatment with VitD_3_ decreased, in a dose-dependent manner, *H. pylori* colonization of the gastric mucosa, especially in wild-type mice, without causing abnormalities in body weight, food intake, or serum calcium or phosphorus levels, indicating that VitD_3_ could efficiently eradicate *H. pylori* infection *in vivo*. The expression of both VDR and CAMP was higher in the gastric tissues of wild-type mice than in those of VDR knockdown mice and was further increased after VitD_3_ administration. Our findings suggest that VitD_3_ could eradicate *H. pylori* through activation of the VDR/CAMP pathway.

As a spiral microaerophilic gram-negative bacterium, *H. pylori* was first isolated from gastric mucosal specimens of a patient with chronic active gastritis, in 1983 (Warren and Marshall, 1983). At present, *H. pylori* has a global infection rate over 50% and is implicated not only in various gastrointestinal diseases but also in other systemic disorders. Regardless of the presence of alarm symptoms, *H. pylori* eradication is considered a necessary measure. The increased resistance to classical antibiotics limits the application of globally recognized triple and bismuth-containing quadruple therapies in developing countries (Malfertheiner et al., 2017). In addition, *H. pylori* has evolved autophagy evasion strategies to support its survival in host cells. Thus, *H. pylori* vacuolating cytotoxin A (VacA) can induce autophagy in several gastric cell lines (Allen et al., 2000) and, together with other virulence factors, has a suppressive role in the autolysosome maturation process. VacA has been found to interfere with lysosome acidification and induce autophagosome formation, leading to massive replication of *H. pylori* in cells (Raju et al., 2012). Therefore, there is an urgent need to develop anti-*H. pylori* agents whose activity would not be compromised by antibiotic resistance or damaging to host cells.

Accumulating evidence indicates that VitD_3_ has anti-inflammatory and anti-microbial effects on *H. pylori* infection and can be used for its eradication. VitD_3_ mainly regulates calcium and phosphate metabolism and the associated physiological processes by activating VDR-dependent signaling. VitD_3_ at high doses has been shown to significantly reduce the risk of autoimmune diseases such as inflammatory bowel disease, type 1 diabetes, and rheumatic disorders (Holick, 2004). Recently, VitD_3_ has been found to protect the gastric mucosal epithelium from *H. pylori* infection, and, by signaling through the VDR, to promote c-Raf/MEK/ERK phosphorylation and prevent apoptosis in *H. pylori*-infected GES-1 cells (Zhao et al., 2022). Interestingly, VitD_3_ has been reported to exert an antibacterial effect through the protein disulfide isomerase A3 (PDIA3) receptor and the downstream STAT3–MCOLN3–Ca^2+^ signaling pathway, thus promoting the recovery of damaged lysosomes and their degradation function, which leads to *H. pylori* clearance (Hu et al., 2019). Therefore, the VitD_3_-PDIA3 pathway emerges as a novel pathway to reactivate autolysosomal degradation, which is critical for VitD_3_ antibacterial activity. Furthermore, VitD_3_ decomposition product 1 and its derivatives can specifically inhibit *H. pylori* (Hosoda et al., 2015; Wanibuchi et al., 2018, 2020). These findings indicate that VitD exerts its anti-*H. pylori* activity *via* various molecular mechanisms.

VitD_3_ biological functions are mediated by VDR, a member of the nuclear hormone receptor superfamily and a ligand-activated transcription factor (Schauber et al., 2007) which is expressed in various tissues, including the intestinal tract, liver, kidneys, muscle, and prostate (Provvedini et al., 1983). VDR is also present in many immune cells, including monocytes, macrophages, natural killer cells, and activated B and T cells. VDR–VitD_3_ signaling is involved in the regulation of cell growth, gene transcription, calcium and phosphorus metabolism, and the activity of the immune system (Amrein et al., 2014). It has been found that VitD_3_ inhibits *H. pylori* through the VDR/CAMP pathway (Zhou et al., 2020). However, the effect of VitD_3_ on *H. pylori* infection *in vivo* has rarely been investigated. Our findings indicate that VitD_3_ reduces *H. pylori* colonization of the gastrointestinal tract through activation of the VDR/CAMP pathway, thus paving the way for the development of novel approaches to *H. pylori* eradication, which could address the problem of antibiotic resistance in this pathogen.

## Data availability statement

The raw data supporting the conclusions of this article will be made available by the authors, without undue reservation.

## Ethics statement

This animal study was reviewed and approved by the Beijing Friendship Hospital Affiliated to Capital Medical University.

## Author contributions

YZ conducted the statistical analysis and drafted the manuscript. CW and LZ critically revised and finalized the manuscript. JY performed the data analysis and interpretation. WY reviewed and edited the manuscript. LL designed the study and performed all experiments. All authors have approved the final version of the manuscript.
